# Food selection, cardiorespiratory fitness and the longitudinal association with adiposity among South African school children: the ExAMIN Youth SA study

**DOI:** 10.1038/s41430-026-01799-y

**Published:** 2026-08-01

**Authors:** Herculina Salome Kruger, Cristian Ricci, Anita Elizabeth Pienaar, Makama Andries Monyeki, Tertia van Zyl, Hannah Ricci, Ruan Kruger

**Affiliations:** 1 Centre of Excellence for Nutrition, North-West University, Potchefstroom, South Africa; 2 Medical Research Council Research Unit for Hypertension and Cardiovascular Disease, North-West University, Potchefstroom, South Africa; 3 African Unit for Transdisciplinary Health Research, North-West University, Potchefstroom, South Africa; 4 Physical Activity, Sport and Recreation Research Focus Area, North-West University, Potchefstroom, South Africa; 5 Hypertension in Africa Research Team, North-West University, Potchefstroom, South Africa

**Keywords:** Risk factors, Developmental biology

## Abstract

**Background:**

The global pandemic of paediatric obesity, unhealthy diets and physical inactivity are important future health challenges. The purpose of this study was to determine 4-year changes in food selection, cardiorespiratory fitness (CRF), and the longitudinal association with the development of adiposity among South African primary school-age children.

**Methods:**

School children aged 5–9 years old in 2017 (*n* = 950) were followed up for four years. Parents indicated selection from healthy and unhealthy food groups in a validated questionnaire. Weight and height of the children were measured, and WHO BMI z-score (BAZ) was calculated. CRF was determined by the 20 m shuttle run test (20-m SRT). Changes in food selection frequency, CRF and BAZ were assessed. The association between frequency of food group selection, age, household income and CRF with 4-year change in BAZ was determined using mixed linear models.

**Results:**

BAZ (*p* < 0.001) and age-adjusted performance in the 20-m SRT of boys (*p* = 0.04) increased at follow-up. The frequency of sugar-sweetened beverages (*p* < 0.001) and milk intake (*p* < 0.02) decreased, while the intake from fast-foods increased (*p* = 0.001). Daily intakes of vegetables and milk were associated with decreases in BAZ. A trend of a positive association was found between frequency of SSB intake and BAZ. CRF showed a strong negative association with BAZ over time (*p* < 0.001).

**Conclusions:**

Higher daily vegetable and milk intakes, as well as higher levels of CRF were protective against increasing adiposity among school-age children. The promotion of healthy eating and measures to improve CRF among school children are important priorities.

## Introduction

The global pandemic of paediatric obesity, unhealthy diets and physical inactivity are future health challenges. The global prevalence of obesity among children increased from 7.1% in studies performed from 2000 to 2011, to 11.3% in studies performed after 2012, according to a meta-analysis. In the same meta-analysis, the prevalence of obesity was significantly higher among those with low physical activity (PA) (12.1%) than in active participants (7.7%) [1]. A systematic review of longitudinal studies among children showed that PA decreased over time across age groups [2]. Low levels of cardiorespiratory fitness (CRF) have been linked to low PA, excessive adiposity, and cardiovascular disease risk markers [3]. Therefore, early detection of childhood overweight and obesity and timely prevention may curb the long-lasting adverse health effects of paediatric obesity [1].

Evidence on the effects of unhealthy foods and beverage intakes among young children in middle- and high-income countries was synthesised in a recent systematic review of longitudinal studies [4]. Sugar-sweetened beverage (SSB) consumption was positively associated with percentage body fat, but showed no association with change in body mass index-for-age z-score (BAZ) at follow-up. The effects of unhealthy food, fat and sugar intakes on increased adiposity were reported in ten studies. Three studies showed a positive association between snack foods and adiposity, whereas seven showed no association, and overall certainty of evidence was low [4]. The authors emphasised the need for prospective studies in low- and middle-income countries on unhealthy foods and SSB, in relation to nutritional outcomes. Such data are needed to make nutritional recommendations aimed at preventing paediatric overweight and obesity [4].

The decline in PA levels in children over time has been linked to a high prevalence of overweight and obesity, as well as low levels of CRF [5]. The latter is regarded as a marker of regular PA, but also of genetic predisposition [6]. CRF was negatively associated with fat mass and mediated a negative relationship between energy intake and fat mass in Spanish children. These results confirm the link between dietary intakes and CRF in maintaining energy balance [7]. Recent studies in South Africa found a high prevalence of both overweight and physical inactivity among children [5, 8, 9], with 50% of children not meeting the recommended 60 min of moderate-to-vigorous PA per day [10]. Urbanisation and the shift away from active transport in rural areas appear to contribute to decreasing PA [11]. The prevalence of paediatric overweight and obesity increases over time in Sub-Saharan Africa [12, 13].

Given the lack of evidence for longitudinal associations between unhealthy food intake, SSB and CRF with adiposity in South African children, the aim of this study was to determine 4-year changes in food selection and CRF and the longitudinal association of these variables with the development of adiposity among South African primary school-age children.

## Methods

This study is part of the longitudinal Exercise, Arterial Modulation and Nutrition in Youth South Africa (ExAMIN Youth SA) study among 5- to 9-year-old children, aimed to determine changes in the prevalence of childhood hypertension and obesity [9, 14]. The study protocol was registered in a clinical trials registry (ClinicalTrials.gov Identifier: NCT04056377), following the Standard Protocol Items: Recommendations for Interventional Trials (SPIRIT) guidelines [15]. Baseline data were collected from 1,103 children during 2017 and 2018. The original sample size calculation indicated that 1,000 children should be included. A total of 950 children were included in the analysis for this study, and 672 were followed up after four years (2021–2022) [14].

### Population and setting

**Fig. 1:**
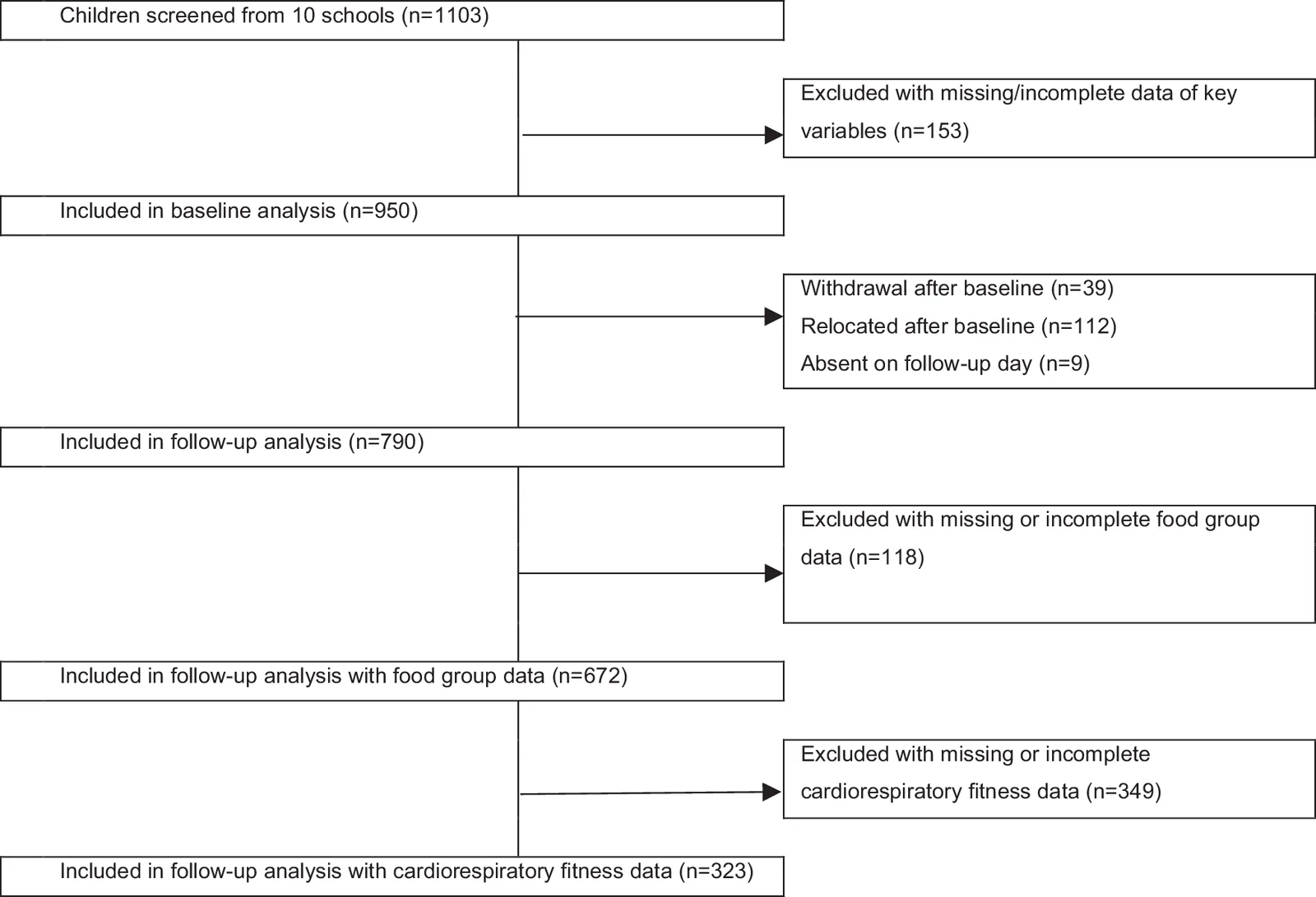
Participant flow diagram for this longitudinal study.

The research team approached the principals of 30 schools and received permission from the provincial Department of Education and principals of 10 urban schools from the Dr. Kenneth Kaunda district in the North West province of South Africa to conduct this research project [9, 14]. Five schools were from quintile three and five schools from quintiles four to five, based on the level of government funding for schools and the area in which the school is located. Quintile one represents communities with the lowest employment rate and literacy, whereas quintile five represents the most affluent [16]. The parents of 1103 children gave informed consent. The only exclusion criterion was children with minor ailments on the day of data collection. Each child < 7 years old signed an informed assent form and those ≥ 7 years signed an informed consent form before measurements, after their parents signed informed consent. After excluding children with missing data for key variables for this part of the study, 950 children were included at baseline. Complete baseline and endline sociodemographic, anthropometric, and food group data were obtained for 672 of these children, while fitness data were available for 323 children. Figure 1 shows the participant flow diagram for this longitudinal study.

The Health Research Ethics Committee (NWU-00091-16-A1) approved the study. A day before baseline data collection, children received an information pamphlet, a General Health and Demographics Questionnaire, and a food group intake questionnaire to take home. Parents had to complete the questionnaires and return them on the day of participation, as reported in detail elsewhere [14].

### Anthropometric measurements and CRF

Weight was taken on a Seca 813 digital scale (Birmingham, United Kingdom). Height was measured barefoot to the nearest 0.1 cm with a Seca 213 stadiometer (Birmingham, United Kingdom) according to the International Society for the Advancement of Kinanthropometry protocol [17]. We calculated body mass index (BMI) and BAZ using the WHO 2006 AnthroPlus growth software [18, 19] to identify underweight (BAZ < -2), normal weight (BAZ -2 to 1) overweight (BAZ > 1 to 2) and obesity (BAZ > 2) [18].

CRF was assessed using the 20-m shuttle run test (20 m SRT), which is a valid and recognised field-based endurance test that shows reliability in children aged six to 16 years. The test involves running back and forth across a 20 m distance [20]. The starting speed is 8.0 km/h, with a 0.5 km/h rise every minute, paced by beeps. A final score is taken when a participant drops out because of exhaustion or cannot cross the 20 m line at the point of the beep for two consecutive 20 m laps [20].

### Food intake questionnaire

Children’s usual dietary intake from 10 food groups was assessed using a validated unquantified food group questionnaire [21]. The questionnaire was developed based on validated questionnaires [22–26], and finalised based on studies from South Africa [22, 27–29] The questionnaire included four healthy food groups, namely fruits, vegetables, milk, meat/fish/poultry/eggs and six unhealthy food groups, tea and coffee with sugar, cold SSB, sweets, salty snacks, cakes and fast foods. The five different responses for frequency of intake were never (0 days), 1–2 days, 3–4 days, 5–6 days, or 7 days per week. Healthy foods were defined as nutrient dense foods containing essential nutrients for child health [22], whereas unhealthy foods were defined as foods high in energy, sugar, salt, and fats, but with low nutrient density. Reliability (item-to-item correlation) delivered a Cronbach’s α of 0.52 for healthy food groups and 0.58 for unhealthy food groups [30].

The same measurements were repeated in 2021-2022, after four years of follow-up, as described in detail elsewhere [9, 31].

### Statistical analysis

Descriptive analyses of the variables were performed. Results were reported as mean and standard deviation for continuous data and counts and percentages for categorical data. The responses of frequency of intake were coded as 0, 1.5, 3.5, 5.5 and 7. Tests were performed for cases with complete data for each test.

Changes in proportions of children within under-/normal weight versus overweight/obesity, as well as within CRF categories were assessed using the chi-square test. BAZ changes over time were assessed using the Wilcoxon signed-rank test. To investigate the effect of the exposures on BAZ over the observational time a random intercept generalized linear mixed model was used. Exponentiated least squares mean at baseline and endpoint were compared by a Wald t-test [32]. The negative binomial distribution was chosen to account for potential overdispersion while a random intercept effect was chosen to consider individual variability. Interaction terms between frequencies of intake from the different food groups, CRF and time (factors) was applied to investigate the longitudinal effect of the exposures on BAZ. Age and household income correlated positively with BAZ at baseline; therefore, the final model was adjusted for these variables.

To determine possible bias introduced by missing data, we compared the baseline data of the group with complete follow-up data for the key variables with those with missing follow-up data. Differences for age, BAZ, and number of laps completed in the 20-m SRT of the two groups were calculated using the independent t-test. Differences between proportions of children per category of school quintile, household income, and food intake frequency were assessed using the Wald test.

All statistical tests were two-tailed with a type-I error rate of 5% for statistical significance. Analysis was performed using SPSS version 30 for Windows (SPSS, Chicago, IL, USA) and SAS version 9.04 (proc glimmix SA, S, Cary, NC, USA).

## Results

**Fig. 2:**
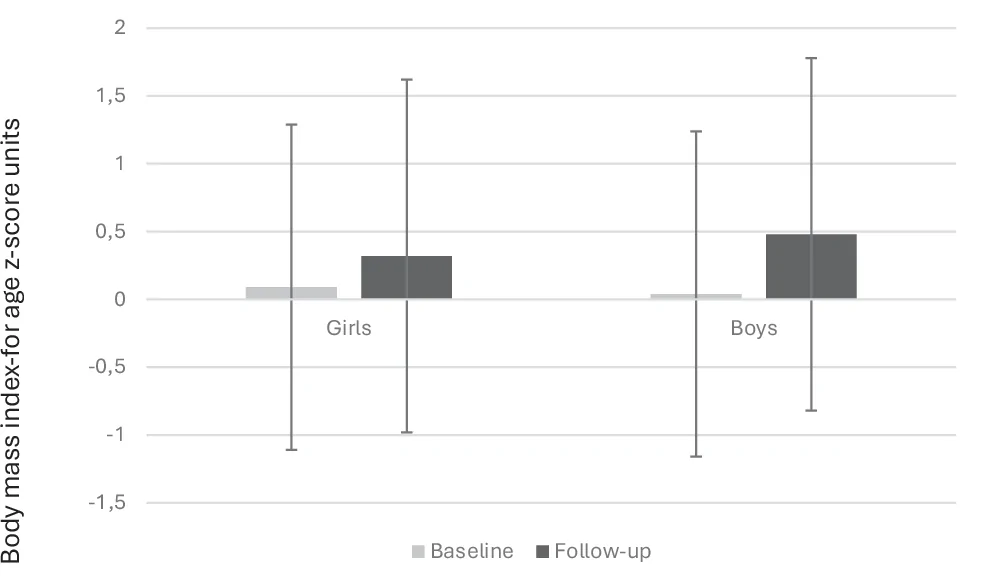
Baseline and follow-up body mass index-for-age z-score of the girls (*n* = 369) and boys (*n* = 303).

**Fig. 3:**
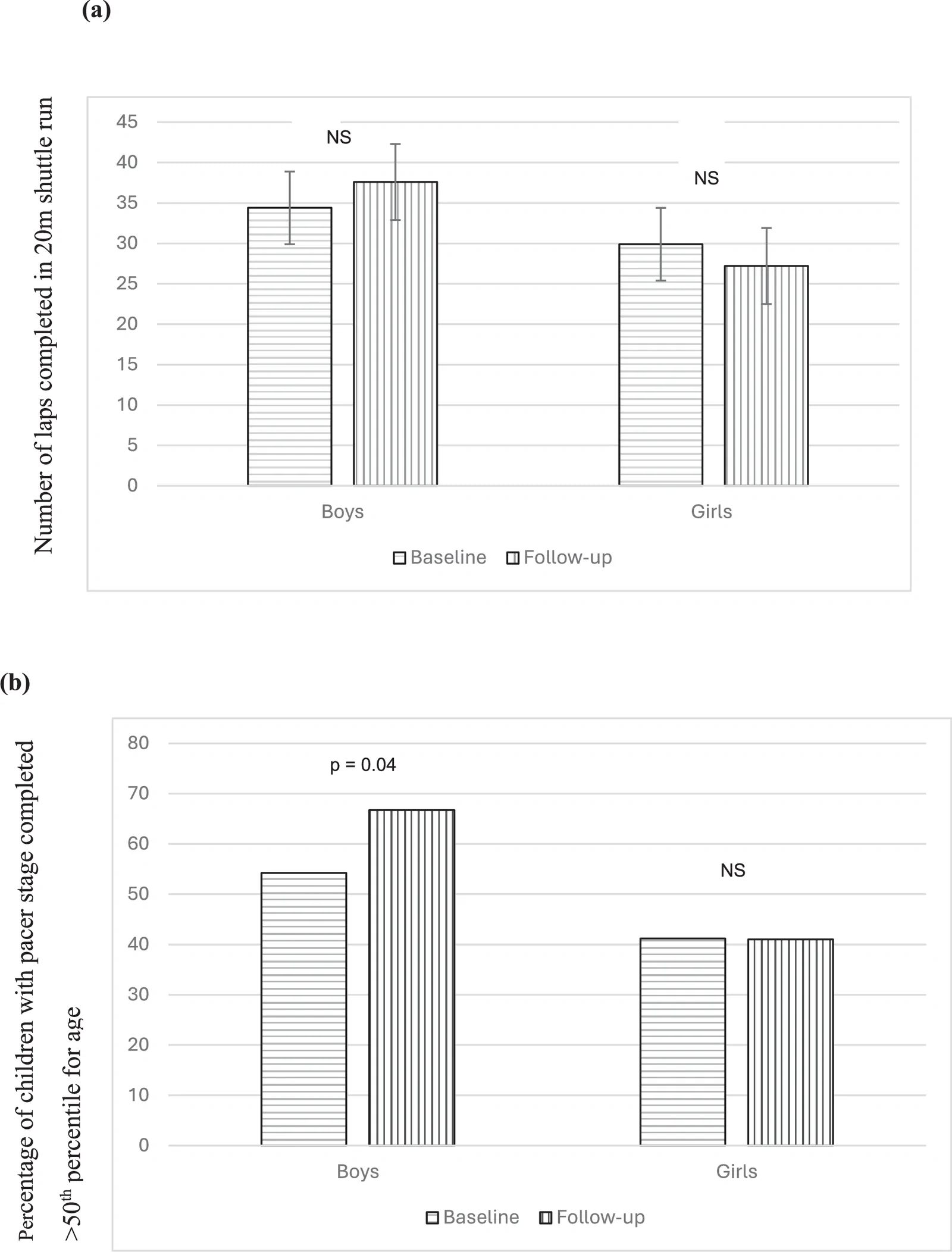
Baseline and follow-up cardiorespiratory fitness of the boys (*n* = 133) and girls (*n* = 188) measured as number of laps completed in the shuttle run test and percentage of children who completed a pacer stage > the 50th percentile for their age. **a** Number of laps completed in the 20 m shuttle run test by boys and girls. **b** Percentage of children with pacer stage completed >50th percentile for age of boys and girls. NS = non-significant change.

**Fig. 4:**
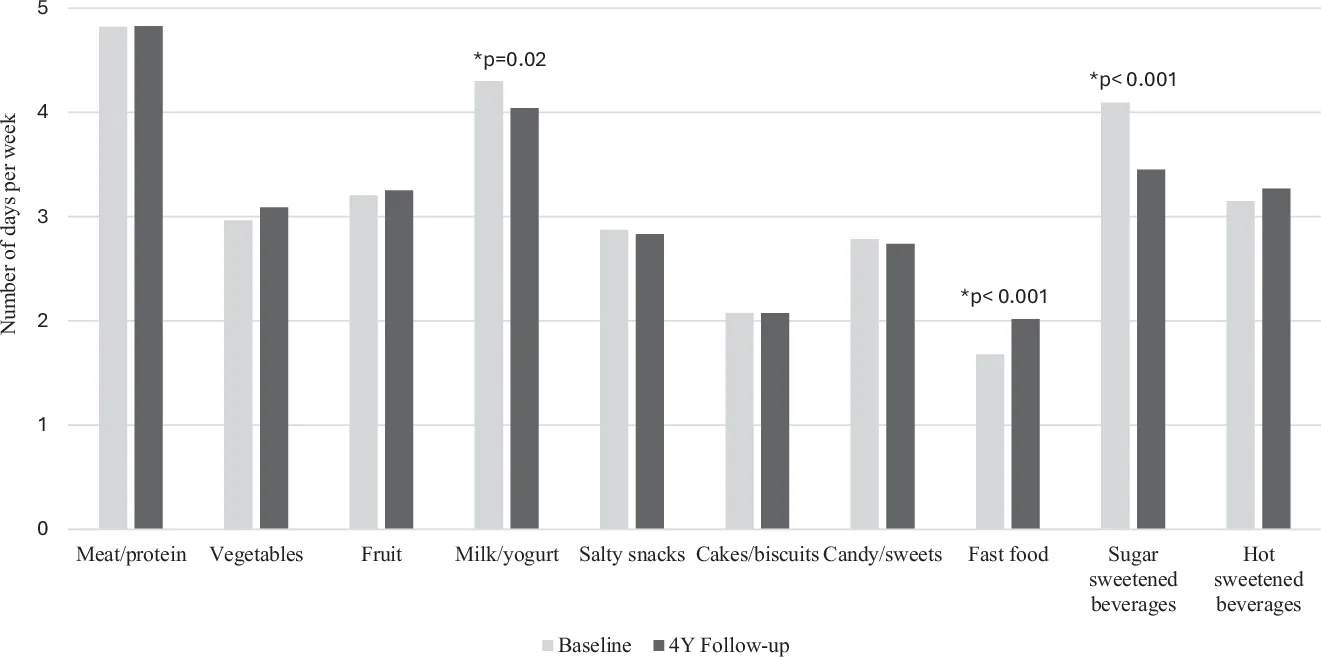
The frequency of intakes (number of days/week) from healthy and unhealthy food groups at baseline and end of the study (least squares estimates from mixed model analysis).

The characteristics of the children at baseline are shown in Table 1. The BAZ (*p* < 0.001, Fig. 2) of all children and age-adjusted performance in the 20-m SRT of boys increased at follow-up (*p* = 0.04, Fig. 3). The percentage of girls who completed pacer stages > the 50^th^ percentile for their age remained unchanged [33]. The frequency of SSB (*p* < 0.001), as well as milk intake (*p* < 0.02) decreased, while the intake from fast foods increased at follow-up (*p* = 0.001). The selection from other food groups remained unchanged (Fig. 4). The prevalence of overweight (14.9–18.2%) and obesity (4.2–14.3%) among the children increased significantly at follow-up (Χ^2^ = 4.44, *p* = 0.04). Table 2 shows a comparison of key baseline variables of the children with complete baseline and follow-up data, with those with missing data at follow-up. No differences between their age, BAZ, and frequency of intakes from key food groups were found. Proportionally more children from quintile four and five schools, than quintile three schools were present at follow-up. The children who were lost to follow-up had lower baseline CRF than those with complete follow-up data.

**Table 1:** Baseline descriptive characteristics of the children (*n* = 950).

Characteristic	Boys		Girls		All children	
	Mean/ frequency	Standard deviation/%	Mean/ frequency	Standard deviation/%	Mean/ frequency	Standard deviation/%
Age, years	7.5	0.93	7.4	0.95	7.4	0.94
Female sex, *n* (%)					507	53.4
*Race:*						
Black, *n* (%)	216	49.8	304	59.0	520	54.7
White, *n* (%)	188	43.3	222	43.1	410	43.2
Asian and mixed race, *n* (%)	9	2.1	11	2.1	20	2.1
*School quintile:* *n* (%)						
Quintile 3, no-school fee schools, government funded	134	30.9	178	34.6	312	32.8
Quintile 4, fee-paying schools, second highest income quintile	176	40.6	212	41.2	388	40.8
Quintile 5, fee-paying schools, highest income quintile	124	28.6	125	24.3	249	26.2
*Anthropometric data:*						
BMI for age z-score	0.03	1.2	0.04	1.1	0.03	1.1
BMI for age z-score categories						
Underweight, *n* (%)	19	4.4	17	3.3	36	3.8
Lean, *n* (%)	331	76.3	399	77.5	730	76.8
Overweight, *n* (%)	63	14.2	78	15.1	142	14.9
Obese, *n* (%)	21	4.8	21	4.1	42	4.4
*Cardiorespiratory fitness*: number of completed shuttle run laps	29.5	14.4	24.9	11.3	27.0	13.0
Shuttle run stage completed	4.0	1.9	3.3	1.4	3.4	1.6

*BMI* body mass index.

**Table 2:** Baseline data of children with complete data versus those with missing data at follow-up for key variables.

Characteristic	Food group data			Cardiorespiratory fitness data		
	Complete data *N* = 672	Missing data *n* = 118	*p*	Complete data *N* = 323	Missing data *n* = 349	*p*
Age (years)*	7.42 ± 0.91	7.47 ± 0.95	0.46	7.53 ± 0.99	7.40 ± 0.88	0.03
BMI for age z-score*	0.02 ± 1.15	0.08 ± 1.12	0.49	−0.02 ± 1.12	0.06 ± 1.15	0.29
Number of pacer laps completed	-	-		29.1 ± 13.6	23.2 ± 11.7	0.001
School quintile 3	220 (32.7%)	55 (46.6%)		156 (48.0%)	91 (25.8%)	
School quintile 4	269 (40.0%)	55 (46.6%)		56 (17.5%)	198 (56.9%%)	
School quintile 5	183 (27.2%)	8 (6.8%)	< 0.001	111 (34.5%)	60 (17.3%)	<0.001
Household income <R1000/month	128 (19.0%)	30 (24.4%)		84 (26.1%)	54 (15.5%)	
Household income R1000-4999/month	135 (20.1%)	37 (31.0%)		77 (23.9%)	68 (19.5%)	
Household income R5000-9999/month	104 (15.5%)	18 (15.5%)		43 (13.4%)	59 (16.9%)	
Household income R10000-20000/month	129 (19.2%)	15 (12.7%)		35 (10.8%)	82 (23.5%)	
Household income >R20000/month	176 (26.2%)	18 (15.5%)	0.05	83 (25.7%)	86 (24.6%)	0.41
Vegetable group intake (days/week) 0	31 (4.6%)	5 (4.2%)		-	-	
1	198 (29.4%)	37 (31.3%)		-	-	
3	265 (39.4%)	42 (35.6%)		-	-	
5	113 (16.9%)	22(18.6%)		-	-	
7	65 (9.7%)	11 (9.3%)	0.93	-	-	
Milk group intake (days/week) 0	26 (3.9%)	3 (2.7%)		-	-	
1	128 (19.0%)	21 (17.5%)		-	-	
3	134 (19.9%)	25 (21.3%)		-	-	
5	150 (22.4%)	26 (22.3%)		-	-	
7	233 (34.7%)	43 (36.1%)	0.69	-	-	

^*^Mean ± standard deviation, independent t-test for differences.

^#^Number (%) in each category, Wald test for independent proportions.

The results of the generalized linear mixed model analyses are shown in Supplemental Table S1. After adjustment for age and household income, selection from the vegetable group 7 times/week vs 0 was associated with a trend of a decrease in BAZ units (estimated reduction = −0.3, *p* = 0.06). Daily intake from the milk group vs no milk showed an estimated reduction of −0.4 BAZ units (*p* = 0.01). In contrast, daily intake of SSB vs. 0 was associated with a trend of increased BAZ of 0.29 units (*p* = 0.07). Unexpectedly, daily intake of cake/biscuits vs 0 was associated with a decrease in BAZ of 0.38 units (*p* = 0.02). The overall frequency of biscuit intake was low, and few children ate biscuits daily (7%). No other associations with food group intakes were found. Each increase in number of pacer laps completed in the 20 m SRT was associated with an estimated reduction of −0.04 BAZ units (*p* < 0.0001) at follow-up.

## Discussion

Overweight and obesity prevalence increased over four years among the primary school-age children, together with an increase in CRF among boys. Although the overall frequency of SSB intake decreased, those children with a daily SSB intake vs no intake showed a trend of higher BAZ. The intake from fast foods increased, but there was no association between fast food intakes and BAZ. Instead, daily selection from the vegetable and milk groups vs none were associated with lower BAZ. The negative association between intake of cake or biscuits with BAZ is difficult to explain. The study was possible underpowered to test for small sample size per group in this specific test, due to the small number of children eating biscuits daily (7%). The number of pacer laps completed in the 20 m SRT test was negatively associated with BAZ over time.

After four years of follow-up almost one-third of the children were in the overweight/obesity category. This prevalence is higher than the national prevalence for this age group, estimated at 12.5% [34]. These results confirm findings of a local study showing increased overweight and obesity prevalence from early childhood to mid-childhood [12]. The marked increase in overweight and obesity could partly be explained by the differences in CRF and the frequency of SSB intake among children in the present study.

Furthermore, it is important to note that baseline data were collected before the onset of the COVID-19 pandemic, while follow-up took place after strict COVID lockdown. Although no measurements for this study could be performed during lockdown, the PA of the children may have decreased. A systematic review of the impact of the COVID-19 lockdown on childhood obesity showed that the abrupt interruption of organized sport led to decreased PA among children [35]. Simultaneously, screen time increased due to distance learning and confinement at home, while fast foods, unhealthy snacks, and SSB consumption increased. Apparently, the higher energy intake and lower energy expenditure may have contributed to the weight gain. This trend is supported by studies that reported significant weight gain during lockdown [35]. For example, in the longitudinal study included in this systematic review, weights and heights of Chinese children were measured before lockdown. After the strict lockdown period 9 months later, the children gained excessive weight [36].

The children’s intake of fast foods increased at follow-up in the current study. Unhealthy food marketing directed at children contributes to a lifelong preference for such foods and influences family food purchases [37]. Therefore, regulation of marketing of unhealthy food to children should be monitored in South Africa [38], where there is currently no legal restriction on the marketing of food to children. The Food and Beverage Advertising Code (2008) and the South African Marketing to Children Pledge (2009) are private sector self-regulatory measures to protect children from unhealthy food and beverage marketing. These codes had no impact in reducing unhealthy food and beverage marketing to children due to weak enforcement [38]. The increasing frequency of consumption of fast foods is of concern and in line with findings from other studies in South Africa, reporting frequent intakes of unhealthy snack foods and SSB across household income groups [27, 39, 40]. The low intakes of fruit and vegetables reported in the current study are in line with other studies among South African children, and are associated with adverse health effects [40].

The age-adjusted number of pacer laps completed remained unchanged at follow-up, but boys performed better at follow-up, in terms of age-related norms [33]. CRF depends on both regular PA and genetic predisposition [6]. Based on this association between PA and CRF, the level of CRF could be an indication of regular PA of children. This assumption is supported by the findings of a study in Spanish children, 9–11 years old [7]. CRF acted as a mediator in the negative relationship between dietary factors and fat mass. Energy and macronutrient intakes showed a negative association with adiposity among children with high levels of CRF [7]. These results confirm our findings that dietary factors, such as daily intakes from the vegetable and milk groups, and CRF are key variables in protecting against excessive adiposity in children.

Physical inactivity among young children remains a major public health problem in many countries [41]. The findings from studies assessing the association between PA and adiposity among children are inconsistent [42, 43]. Most of these studies were conducted in high-income countries. A few studies in South African children focused on preschool children and adolescents [44, 45], while studies among primary school-age children had inconsistent results [46]. This may be due to the challenges related to accurate measurement of PA in this age group [5, 8]. Studies in primary school-aged South African children indicated that the children’s PA level correlated negatively with percentage body fat, based on prediction equations [46] and BIA [5]. Another study found significant positive associations between body mass and sedentary time among urban South African school children. The same study indicated an age-related decline in PA and increase in sedentary time among school-age children, linked to an increased obesity prevalence [47]. These results are in line with the results of the current study, indicating that PA and CRF may prevent excessive weight gain over time.

The increasing obesity prevalence among children should be addressed. Early detection and prevention of childhood obesity is important to improve their future health and to relief the growing burden of adult comorbidities. Poor diets and physical inactivity are modifiable risk factors that could be addressed in school-based interventions [48]. According to data from two Cochrane reviews, PA interventions in children were more effective in preventing obesity compared to nutrition interventions [49, 50]. The most effective combination was school setting interventions with individualised high-intensity PA of short duration and behaviour modification [49]. A systematic review of the effectiveness of school-based obesity prevention interventions on the health behaviour of children showed a positive impact on fruit and vegetable intakes, PA and decreased SSB intake [50].

Limitations of this study include the assessment of CRF using only a field-based test in a subsample of children due to logistical constraints. Due to challenges in assessing the PA of the children, the 20 m SRT was used to determine CRF, as a proxy for PA. The food groups questionnaire data were based on recall and reported by the parents of the children. Recall bias could have had an impact on the validity of these data. The study participants attended South African quintile groups three to five schools, indicating medium to high employment rate and literacy of the communities. Quintiles one and two represent the poorest schools [16], and these participants were excluded from this study due to logistical considerations. Furthermore, proportionally more children from quintile three schools were lost to follow-up. The results may therefore not apply to children from the lowest socio-economic status groups in South Africa. Despite these limitations the strength of the study was that it was the first longitudinal study of intakes from healthy and unhealthy food groups in a relatively large sample of primary school-aged South African children over a 4-year period, and the association with adiposity.

In conclusion, daily vegetable and milk intakes, and higher levels of CRF were protective against increasing adiposity among school-age children, whereas daily SSB intake showed a trend of a positive association with BAZ. These findings indicate that promotion of healthy eating and measures to improve CRF should be prioritised among school children.

## Supplementary information

Supplementary Table S1

## Data Availability

The datasets generated and analysed during the current study are available from the corresponding author on reasonable request.
