# Supplementary material for: Food selection, cardiorespiratory fitness and the longitudinal association with adiposity among South African school children: the ExAMIN Youth SA study
Source: Eur J Clin Nutr. 2026 Aug 1;80(10):1025–32. doi: 10.1038/s41430-026-01799-y (PMC13690588; doi:10.1038/s41430-026-01799-y)
Supplement: Supplementary file 1 [file 41430_2026_1799_MOESM1_ESM.pdf]

Supplementary Table S1. The results of the generalized linear mixed model analyses of the effects of food group intakes and cardiorespiratory fitness on BAZ over the observational time of four years

**Exposure comparison**	**Outcome : Change in BAZ units (95% CI)**	**P**
Model without adjustment		
Frequency of vegetable intake: daily versus never	-0.36 (-0.69, -0.03)	0.03
Frequency of milk group intake: daily versus never	-0.47 (-0.79, -0.13)	0.01
Frequency of SSB intake: daily versus never	0.33 (0.02, 0.64)	0.04
Frequency of cake/biscuits intake: daily versus never	-0.41 (-0.72, -0.09)	0.01
Each increase in number of pacer laps completed in the 20-m SRT	-0.04 (-0.08, -0.02)	<0.0001
Model with adjustment for age and household income		
Frequency of vegetable intake: daily versus never	-0.32 (-0.67, 0.02)	0.06
Frequency of milk group intake: daily versus never	-0.44 (-0.78, -0.08)	0.01
Frequency of SSB intake: daily versus never	0.29 (-0.03, 0.61)	0.07
Frequency of cake/biscuits intake: daily versus never	-0.38 (-0.72, -0.06)	0.02
Each increase in number of pacer laps completed in the 20-m SRT	-0.04 (-0.07, -0.01)	<0.0001

BAZ, body mass index -for-age z-score; CI, confidence intervals; P, level of significance; SSB, sugar sweetened beverages; 20-m SRT, 20-m shuttle run test
